# Differences in Sexual Behavior and Partner Notification for Sexually Transmitted Infections Between the Out of School Youth and University Students in a Peri-Urban District in South Africa—A Cross-Sectional Survey

**DOI:** 10.3389/fpubh.2022.793702

**Published:** 2022-06-22

**Authors:** Mathildah Mokgatle, Sphiwe Madiba, Naomi Hlongwane

**Affiliations:** ^1^Department of Biostatistics, School of Public Health, Sefako Makgatho Health Sciences University, Ga-Rankuwa, South Africa; ^2^School of Transdisciplinary Research and Graduate Studies, College of Graduate Studies, University of South Africa (UNISA), Pretoria, South Africa; ^3^Department of Environmental and Occupational Health, School of Public Health, Sefako Makgatho Health Sciences University, Ga-Rankuwa, South Africa; ^4^School of Public Health, School of Health Care Sciences, Sefako Makgatho Health Sciences University, Ga-Rankuwa, South Africa

**Keywords:** risky sexual behaviors, STI/HIV, university students, out of school, partner notification, South Africa

## Abstract

The increase in sexually transmitted infections (STIs) in young people is a public health concern. Among those in university and out of school, different contextual factors contribute to their risky sexual behavior and increased susceptibility to STIs and HIV. There are limited comparative studies examining risky sexual behavior and partner notification (PN) between these two groups, particularly in South Africa. We investigated sexual behaviors, self-reported STI diagnosis, health seeking behavior, and preferred PN methods of university students and out of school youth. A descriptive cross-sectional survey was used using convenient sampling to select 917 students across five health sciences universities and through periodic sampling 699 out of school youth were selected from two main local shopping centers in South Africa. Descriptive statistics, bivariate and multivariable logistic analysis were performed using Stata IC version 14. More university students (71.7%) than out of school youth were in casual relationships (28.3%), with half of out of school youth being in steady relations (50.2%). Moreover, university students (65.7%) used a condom in the past 6 months compared to their counterparts (34.3%). Of the 124 youth who were diagnosed with STI in the past 12 months, majority (*n* = 106, 85%) were out of school youth. The probability of notifying a partner about a STI infection was 82% among university students compared to their counterparts (*p* = >0.05). The odds of notifying a partner was 1.79 times more for those having multiple sexual partners than those who had only one partner. Both groups preferred a face-to-face STI disclosure with partner; however, more university students (67%) preferred SMS notification than PN referral slips as compared to out of school youth (42%). Both the university students and the out of school youth engaged in risky sexual behaviors. Both groups preferred face-to-face and clinic SMS partner notifications, even though university students were in the majority. There is a need for developing health promotion scripts on disclosing STIs to sexual partners to empower the majority of the youth who prefer face-to-face PN over the prescribed methods.

## Introduction

Sexually transmitted infections (STI) are of great public health concern and are key epidemiological markers for unprotected sex (1). Globally, 376 million STI diagnoses were reported amongst persons aged 15–49 with a daily infection rate of 1 million (1). STIs are the largest burden of disease in the African region with South Africa accounting for 18% of the global infection rate (2). The HIV prevalence amongst young people presenting with STI syndrome is at an estimated 19% of the general South African population and the co-infection rates reflect an epidemiological synergy that exists between STIs and HIV. This synergy puts young people at the center of the HIV epidemic (3–7).

Young people's risky sexual behavior has been well-documented and studies have reported on the public health initiatives geared toward curbing them. Furthermore, youth in South Africa continue to engage in risky sexual practices, making this the key reason for their vulnerability to contracting STD's and HIV (8–10). Studies looking at the transition to adulthood in the context of AIDS highlighted that the following factors contribute and form the high risky sexual behavior among young people; early sexual debut, age of sexual partner, multiple partners, inconsistent condom use, currently in school and peer influence (11, 12, 61).

Studies show that students in higher learning institutions form part of this vulnerable age group and they further have added contextual factors that contribute to their increased susceptibility to STIs and HIV (13–16). Moser and colleagues indicate that a notable contextual factor is that university students have begun experimenting sexually and, because they live and interact with a large number of young people, this further perpetuates sexual activities that are not monogamous (17). In addition access and consumption of high levels of alcohol, increased sexual opportunities, limited sexual health services in learning institutions and being free of moral surveillance have been reported as associated contextual factors (18). A survey conducted by The Higher Education HIV and AIDS Programme (HEAIDS) among South Africa's institutions of high learning including technical and vocational education and training (TVET) reports that ~61% of students at TVET colleges had a sexual partner, with 55% reporting consistent condom use (19). However, some studies are of the view that attitudes to condom use are negative and that condom use was only practiced if the partner requested it (20–22).

Little is known about the sexual behaviors of out of school youth who in this study can be described as not attending school, not finished school (including elementary school) or any college or post-secondary school course, and not working who are exposed to different social and contextual settings as those who are within institutions of higher learning (9, 23–26, 59). According to STATS SA which is the national statistical service of South Africa, the out of school youth account for 34% (3.2 million) of the population group who are unemployed and not within the education system (60). An earlier study on out of school youth reported that 65% had sexual partners with 33% having sex with non-regular partners. A substantial number of out of school youth—especially males—had unsafe sexual intercourse and this included sex with female sex workers which, over and above the abuse of substances, was a great contributing factor to risky sexual behavior (27). There are thus notable difference in factors that contribute to the risky sexual behaviors among out of school youth and university students.

Comparative studies done have noted that out of school youth were engaged in more risky sexual behaviors than in school youth, and had an earlier sexual debut than those in school and this can be due to in school youth being more exposed to formalized HIV/AIDS information and sexual education than their counterparts (26, 28). This reflects that there are differences in factors that contribute to the risky sexual behavior amongst out of school youth and in school youth.

Within low to middle income countries like South Africa, symptomatic STIs have been treated by syndromic management since 2009; however, most STIs are asymptomatic and as a result go untreated (29, 30). With the burden of asymptomatic infections being highlighted amongst young people, this hampers the prevention and management of STIs. In light of the fear of stigma and rejection, young people are unlikely to report symptoms and to notify partners. South Africa's current partner notification protocol entails prescribing a syndromic management regime, offering education on STI/HIV and patient counseling (31, 32). Embedded in this protocol is the patient-initiated PN by the use of notification and referral slips where index cases often bear the sole responsibility for notifying their partners (33, 34). Research shows that there are significant barriers to this standard of practice such as under reporting of sexual partners, limited slips from health care workers and lack of follow up strategies, as well as time dedicated to counseling (33, 35). Regardless of the strategies mentioned, the rates of partner notification remain low with a lack of a standard preferred method (34, 36).

PN and treatment are essential components of STI management, and when implemented effectively can prevent index patients from reinfection from untreated sexual partners and reduce the burden of curable STI in this population group (37, 38). However, the implementation of STI PN remains limited in developing countries such as South Africa due to poor infrastructure for the diagnosis and management of STI, a lack of resources and social stigma (39, 40). Furthermore, little is known about partner notification practices among young peoples in general but more specifically those in university and out of school. Studies report that stigma, lack of STI knowledge, expectation of negative response from partner and fear of blame were some of the reasons for not notifying a partner of STI. In addition, those who did notify their partners experienced a negative emotional experience. STI partner notification thus remains an obstacle among youth (41, 42).

Risky sexual behaviors among young people and their susceptibility to STI's and HIV have been well-documented (7, 8, 10, 16). However, in light of the social and contextual factors associated with risky sexual behavior there are limited studies that compare young people who are in different contexts in South Africa in particular. In response to the dearth of data, this study aimed to explore and compare sexual behavior, self-reported STI and partner notification practices, and preferences among out of school youth and in university students. Since risky sexual behaviors are linked with an increased risk of HIV infection, understanding risky young people's sexual behaviors across the two groups is crucial. As such, we explored their risky sexual behavior, their self-reported STI prevalence and their partner notification practices. Failure to inform sex partners of exposure to STIs increases the risk of STI transmission to other sexual partners who remain asymptomatic (32). Knowing the difference and similarities between these two groups will assist in tailoring peer group specific educational materials and messaging which will contribute toward reducing risky behaviors and STI transmission among the youth.

## Methods

### Study Setting and Population

An institution based and a community based cross-sectional study was conducted from March to November 2018. The study participants were the university students and the out of school youth from the sub-district of Tshwane, Gauteng Province. The out of school youth were from a township, which is a peri-urban area within the vicinity of the university. University students were enrolled in the undergraduate programmes of a health care sciences university, whereas the out of school youth were recruited from two shopping malls in the sub-district. The rationale for the comparative analysis was based on the premise that both groups shared a context of living within the same district and the students were bound to interact with the community services as they accessed economic, social, and other recreational services from the establishments in the district.

The sample age ranged from 18 to 35 years of age, an age range that was informed by the South Africa's National Youth Commission Act 1996, which defines youth as those from ages 14 to 35 years. The descriptors for out of school youth were not attending school, had not finished school (including elementary school) or any college or post-secondary school course, and were not working at the time of data collection (43).

### Study Design and Sampling Procedures

This paper is one of a series of manuscripts from a large formative evaluation study on the acceptability and feasibility of implementing STI provider-initiated partner notification using SMS. The methodology of the main study is described in detail in the article by Mokgatle and Madiba (35). University students and the out of school youth were identified as some of the high-risk populations in the main study.

A descriptive, cross-sectional design was used to carry out the surveys on sexual behaviors, condom use and partner notification for sexually transmitted infections among the undergraduate students and the out of school youth.

The university student sample included undergraduate students from year 1 to year 5 of study, since the degree programmes in the health sciences university range from 3 to 6 years long.

The sample size of the students was *N* = 917, calculated at 95% confidence level and 5% confidence interval is estimated at 180 students per year of study. For the 5 year levels the total sample was *N* = 900 students and *N* = 17 additional students were included due to convenient sampling of the students in the classrooms. The sample size for out of school youth was *N* = 699. This was a sample of convenience as the out of school participants were accessed using a periodic sampling technique at two main local shopping centers where the out of school youth spent time during the day.

### Data Collection Tool and Procedures

Data was collected using a structured self-administered questionnaire prepared in English among university students and used the same questionnaire translated to a local language for out of school youth,. To maintain the privacy of participants, seats were arranged far apart in the classrooms for university students, while closed gazebos with folding tables and chairs were erected at the malls for the out of school youth.

### Measures

The data were collected through a structured self-administered questionnaire. The original tool that was used to collect data for the main study consisted of sociodemographic, sexual relationships and behaviors using a 39 item scale. An 18 item scale was used to measure condom use and HIV risk perception, a 16 item scale to measure knowledge of STI and awareness of partner notification, an 11 item scale was used to assess the practice and behavior around use of partner notification slips in a subgroup of those who reported to have been diagnosed with STIs, a 11 item scale to measure the acceptability and perceived intentions to use a referral slip and SMS partner notification. For purposes of this study, we focused our measures on comparable demographics relevant to students and out of school youth sociodemographic, sexual behavior and condom use and the questions included condom use in the last sexual act, unprotected sex, number of sex partners, concurrent partnership, and transactional sex. To assess the level of risk perception, a five-item three-scale Likert scale was employed. The students were asked how worried they were of getting HIV and the chance of contracting STIs. The response was categorized as 0 = not likely to contract the disease and was categorized as having low risk perception, and 1 = likely to contact the disease as having high risk perception.

The students were asked if they were diagnosed with STIs in the last 12 months and the common symptoms they experienced. The participants who gave an affirmative response to STI diagnosis were further asked about their health-seeking behavior and partner notification practice.

The partner notification questions were compiled in a five-item measure. The questions that covered intentions to notify sexual partners if they had an STI were assessed using seven items asking whether they would notify their partner if they had an STI and the preferred partner notification method. Responses were categorized as “Yes,” “No,” and “Not sure.” For prior partner notification experiences, students were asked whether they had ever informed a sexual partner that they had been diagnosed or treated for an STI, and asked them whether a sexual partner had ever informed them of an STI.

### Data Collection

We used a self-administered standardized English questionnaire for data collection among university students and used the same questionnaire translated to a local language for out of school youth, even though the out of school youth were given an option of either of the questionnaires. The questionnaires were administered by trained research assistants who distributed the questionnaires to the target population and checked them for completeness. Three days of intensive training were given to five data collectors to administer the questionnaire. The questionnaire was developed by referring to previous tools and constructs obtained from the review of the literature on STIs, risky sexual behaviors, and partner notification (25, 38, 44).

### Ethical Considerations

The Research Ethics Committee of Sefako Makgatho Health Sciences University (SMUREC/H/284/2015: IR) reviewed the protocol and gave an ethical clearance. The relevant university authorities and shopping center managers granted permission and offered their facilities to conduct the study. Due to the nature of convenient sampling for the out-of-school youth in the shopping centers and for the university students in the class rooms, there was potential for cohesion of participants to participate in the study. Cohesion was controlled by the process of administering and obtaining informed voluntary consent from all university students and the out of school youth. The informed voluntary consent included the right to withdraw from the study without any preconditions. For all the participants that were sampled and requested to participate in the study, there were no refusal to participate or withdrawals during questionnaire administration. For anonymity, no identifying information was collected and the data file was password protected, with access limited to the lead investigator.

### Data Analysis

Data analysis using a comparative analysis between university students and the out of school youth was performed using Stata Statistical Software: Release 14 (45).

Initially, descriptive analysis using bivariate analysis for the two groups was conducted to describe the participants' characteristics and sexual relationship status. A Chi-square test was performed to determine the differences between the two groups—university and out of school youth.

We compared the two groups with regard to socio-demographics, self-reported STIs, partner notification patterns, and the choice of partner notification method. University students were the reference group since they are in a controlled campus environment, they were occupied with relevant age related activities of studying and recreation as youth compared to their out-of-school counterparts.

A binary logistic regression model was used to determine the association between sexual behavior and participant group (i.e., being a University student or Out of school youth). For comparing University student to the out-of-school youth, we build a logistic regression model from eight (8) variable that were statistically significant (*p*-value <0.050) at the Chi-Square test, and the outcome variable was sexual behavior. The variables included in the model were, number of sexual partners in the past 12 months, had transactional sex in the past 12 months, condom use in the past 6 months, condom use in the last sexual encounter, refused sex without condom, suggest use of condom to partner, discuss HIV testing with partner, and worrying about HIV infection.

Furthermore, multivariate logistic regression was used to analyse notifying a partner for having been infected with STIs and the associated factors. In this model for assessing partner notification, the two groups were combined into one sample. The outcome variable was partner notification and eight (8) variables included in the model were, age, sex, educational group, number of sexual partners in the past 12 months, number of years in a relationship, had transactional sex in the past 12 months, condom use in the past 6 months, and HIV testing with a partner. The adjusted and unadjusted odds ratios (ORs) and the 95% confidence interval (*p*-value <0.05) were used to describe the strength of the association between the dependent and independent variables.

## Results

### Demographic and Behavioral Characteristics of Participants

The distribution of the youth by their various socio-demographic characteristics and relationship details are shown in Table 1. The total sample consisted of 1,616 participants. For data analysis, all the participants who reported not to be in a sexual relationship and were not sexually active (*N* = 45) at the time of data collection were excluded from the data analysis for this paper. We therefore analyzed data on a sample of 1,571 participants, of which *N* = 904 were university students and *N* = 667 were the out of school youth. The median age of the youth was 21 years (IQR ± 2 years), most (*n* = 1,206, 77.4%) were female, the majority (999, 81.7%) were in steady relationships, and 54.6% (*n* = 601) were in a relationship that was between 1 and 4 years' duration. There was a significant difference in the relationship status between the groups, with almost a third (*n* = 160, 71.7%) of the university students in casual relationships compared to the out of school youth (*p* < 0.001). More out of school youth were in relationship for longer, and the difference was significant (*p* < 0.001).

**Table 1:** Demographic characteristics of the youth by group.

**Variables**		**Frequency (percentage)**		
	**Total (*n* = 1,571)**	**University students (*n* = 904)**	**Out of school youth (*n* = 667)**	***P*** **-value**
**Sex**				
Female	1,206 (77.4)	631 (52.3)	575 (47.7)	<0.001^*^
Male	352 (22.6)	270 (76.7)	82 (23.3)	
**Age, years**				
18–24	1,397 (89.4)	820 (58.7)	577 (41.3)	0.001^*^
25–35	166 (10.6)	76 (45.8)	90 (54.2)	
**Relationship status**				
Casual partner	223 (18.2)	160 (71.7)	63 (28.3)	<0.001^*^
Steady partner	999 (87.8)	498 (49.8)	493 (50.2)	
**Years in relationship**				
<1	277 (25.2)	188 (67.9)	89 (32.1)	<0.001^*^
1–4	601 (54.6)	297 (49.4)	304 (50.6)	
≥5	223 (20.2)	56 (25.1)	167 (74.9)	

* *Significant at p < 0.05*.

### Risky Sexual Behaviors of University Students and Out of School Youth

Disaggregating data of the youth by their risky sexual behaviors revealed that in the past 12 months 29.1% (*n* = 343) of the participants in the two groups had sex with more than one partner, of which 55.7% were the out of school youth, and 44.3% were university students. The difference was statistically significant (*p* = 0.03). Concerning transactional sex in the past 12 months, out of school youth accounted for 58.3% of those who had transactional sex compared to 41.7% of university students. These differences were statistically significant at *p*-value < 0.05.

With respect to the use of condoms, 607 (45.7%) of the participants did not use condom during their last sexual intercourse, more (56.0%) of the out of school youth compared to their counterparts (44.0%). These differences were statistically significant at *p*-value < 0.001. On the contrary, more university students used a condom in the past 6 months compared to their counterparts (65.7 vs. 34.3%). This difference was statistically significant at *p*-value of <0.001. Moreover, more university students compared to out of school youth did not carry condoms (67.4 vs. 32.6%; *p* = 0.000), and over half of them (60.6%) were unlikely to refuse sex without a condom compared to 39.4% of out of school youth. The differences were statistically different (*p* = 0.006).

Considering the synergy between HIV and risky sexual behavior, the results revealed that more (54.6%) out of school youth had a low HIV risk perception as compared to university students (45.4%), whereas more of the university students, 66.2% compared to 33.8% of the out of school youth, would not discuss HIV testing with a sexual partner (*p* < 0.001) (Table 2).

**Table 2:** Risky sexual behavior.

**Variables**		**Frequency (percentage)**		
	**Total (*n* = 1,571)**	**University students (*n* = 905)**	**Out of school youth (*n* = 667)**	***p*** **-value**
**Number of sexual partners in the past 12 months**				
One	836 (70.9)	430 (51.4)	406 (48.6)	0.03^*^
Two or more partners	343 (29.1)	152 (44.3)	191 (55.7)	
**Had transactional sex in past 12 months**				
No	1,417 (96.7)	791 (55.8)	626 (44.2)	0.05^*^
Yes	48 (3.3)	20 (41.7)	28 (58.3)	
**Condom use last 6 months**				
Never	219 (17.3)	91 (41.5)	128 (58.5)	< 0.001^*^
Sometimes	539 (42.7)	207 (38.4)	332 (61.6)	
Always	505 (40.0)	332 (65.7)	173 (34.3)	
**Used condoms in last sexual encounter**				
No	607 (45.7)	267 (44.0)	340 (56.0)	< 0.001^*^
Yes	721 (54.3)	411 (57.0)	310 (43.0)	
**Carry condoms when needed**				
Yes	567 (39.6)	185 (32.6)	382 (67.4)	< 0.001^*^
No	865 (60.4)	583 (67.4)	282 (32.6)	
**Refuse sex when no condom**				
Unlikely	343 (23.7)	208 (60.6)	135 (39.4)	0.006^*^
Likely	1,107 (76.3)	577 (52.1)	530 (47.9)	
**Worried about getting HIV/AIDS**				
Not worried at all	568 (36.9)	258 (45.4)	310 (54.6)	< 0.001^*^
Worried	308 (20.0)	175 (56.8)	133 (43.2)	
Very worried	663 (43.1)	445 (67.1)	218 (32.9)	
**Can discuss HIV testing with partner**				
No	317 (21.6)	210 (66.2)	107 (33.8)	< 0.001^*^
Yes	1,152 (78.4)	632 (54.9)	520 (45.1)	
**Chances of HIV testing with partner**				
Unlikely	393 (27.1)	239 (60.8)	154 (39.2)	0.07
Likely	1,060 (72.9)	589 (55.6)	471 (44.4)	

* *Significant at p < 0.05*.

### STI Related Factors

**Figure 1:**
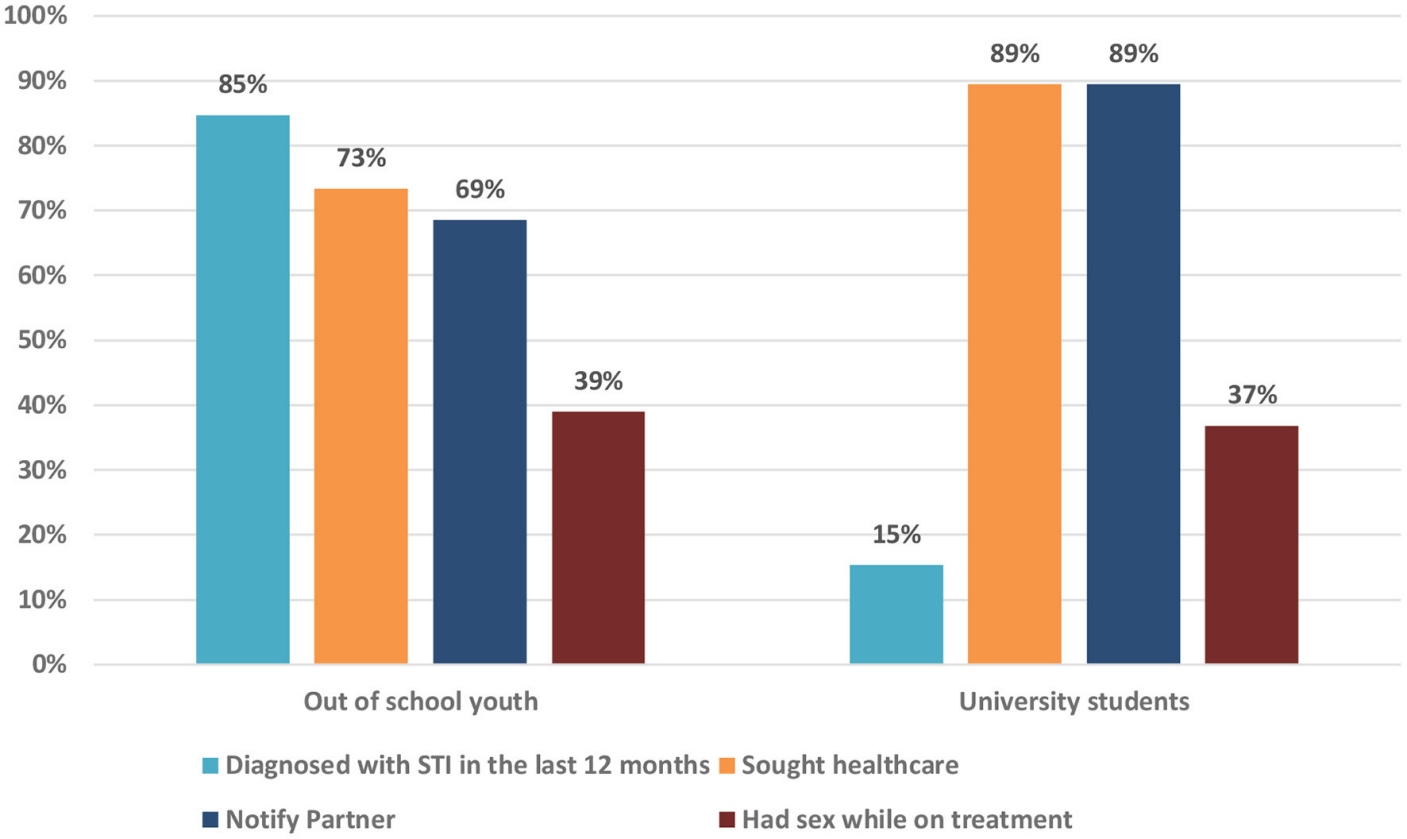
Distribution of youth diagnosed with an STI in the past 12 months.

Of the 124 youth who were diagnosed with STI in the past 12 months, 85% were out of school youth compared to 15% of the university students. Despite a lower rate of STI diagnosis among university students, more (89%) received treatment as compared to the out of school youth (73%), and a higher rate of PN was reported by university students (89%) compared to their counterparts (69%). Concerning the sexual behavior after STI diagnosis, both groups reported to have had sex while taking STI treatment (Figure 1).

### Choice of Partner Notification Method

**Figure 2:**
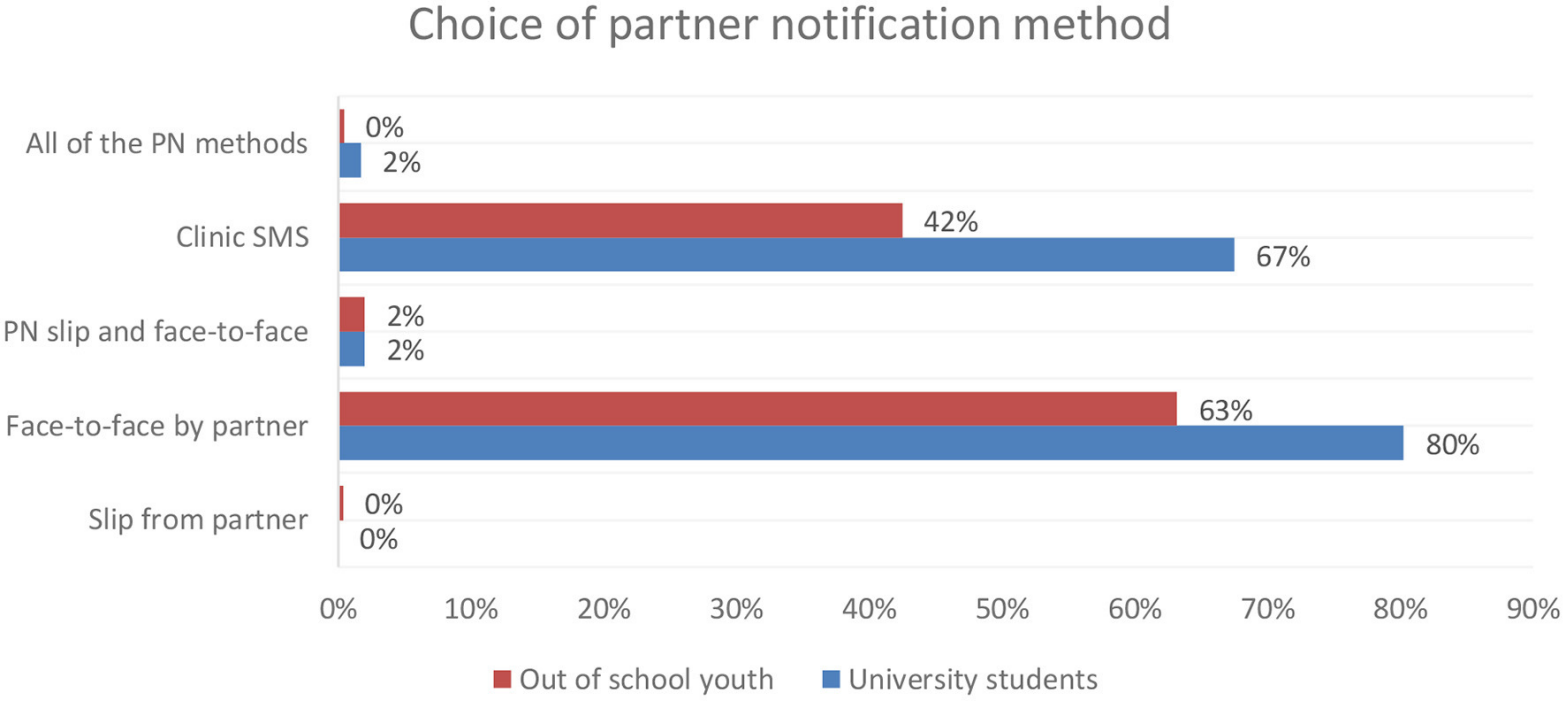
Choice of partner notification if partner is diagnosed with an STI.

The majority (73%, *n* = 94) of the youth in both groups would notify their partner if they were infected with STI. Although both groups preferred their partner to notify them face-to-face if they were diagnosed with an STI, more university students preferred face-to-face notification than their counterparts did (80 vs. 63%). More of the university students preferred to receive an SMS from the clinic notifying them of an STI diagnosis (67 vs. 42%; Figure 2).

### Perceptions About Partner Notification

Being able to deliver a PN slip was significantly different between the groups (*p* < 0.01). More (62.1%) of the out of school youth would not deliver a PN slip compared to 37.9% of the university students. Despite more university students being prepared to deliver a PN slip, the majority (70.3%) said it was not an easy task. The mode of how the PN slip was delivered was significantly different across the two groups (*p* > 0.01). Over half (60.2%) of university students preferred an SMS over the PN slip currently used for PN than out of school youth (39.8%), and more (56.7%) university students were of the opinion that an anonymous SMS worked better than a PN slip compared to their counterparts (43.3%). A significant difference was observed for PN perceptions for both groups (*p* > 0.01) (Table 3).

**Table 3:** Partner notification practices of STI.

		**Frequency (percentage)**		
	**Total (*n* = 1,571)**	**University students (*n* = 904)**	**Out of school youth (*n* = 667)**	***p*** **-value**
**Can deliver PN slip to partner**				
No	528 (35.7)	200 (37.9)	328 (62.1)	< 0.001^*^
Not sure	293 (19.8)	240 (81.9)	53 (18.1)	
Yes	658 (44.5)	372 (56.5)	286 (43.5)	
**Can use PN slip delivered by partner**				
No	50 (3.4)	30 (60.0)	20 (40.0)	< 0.001^*^
Not sure	124 (8.4)	101 (81.4)	23 (18.6)	
Yes	1,308 (88.3)	685 (52.4)	623 (47.6)	
**Easy to deliver PN slip to partner**				
Not easy	526 (35.4)	370 (70.3)	156 (29.7)	< 0.001^*^
Not sure	177 (11.9)	139 (78.5)	38 (21.5)	
Easy	781 (52.6)	311 (39.8)	470 (60.2)	
**Prefer SMS slip over PN slip**				
No	58 (5.9)	30 (51.7)	28 (48.)	< 0.001^*^
Not sure	114 (11.7)	88 (77.2)	26 (22.8)	
Yes	805 (82.4)	485 (60.2)	320 (39.8)	
**Would an anonymous SMS work better than PN slip**				
No	548 (37.1)	261 (47.6)	287 (53.4)	< 0.001^*^
Not sure	156 (10.5)	114 (73.1)	42 (26.9)	
Yes	775 (52.4)	439 (56.7)	336 (43.3)	

* *Significant at p < 0.05*.

### Sexual Behaviors Associated With the Group Characteristics

Table 4 reflects a binary and multivariate logistic regression analysis that was performed to find the association between sexual behavioral factors of the groups. On bivariate analysis, seven predictor variables were found to have an association with the groups. Multivariate logistic regression was done to identify the independent effect of the variables by controlling the confounding effect of other variables. The strongest associations were number of sexual partners, overall condom use, condom use in last sexual encounter, always carrying condom, refuse condomless sex, discuss HIV testing with partner, and being worried about contracting HIV.

**Table 4:** Bivariate and multivariable logistic regression analysis of sexual behaviors associated with the group characteristics.

	**University students *n* (%)**	**Out of school *n* (%)**	**UOR (95% CI)**	**AOR (95% CI)**
**Number of sexual partners in the last 12 months**				
One	430 (51.4)	406 (48.6)	Ref	Ref
Two or more partners	152 (44.3)	191 (55.7)	0.75 (0.58–0.97)^*^	0.54 (0.38–0.80)^*^
**Had transactional sex in past 12 months**				
No	791 (55.8)	626 (44.2)	Ref	Ref
Yes	20 (41.7)	28 (58.3)	0.56 (0.31–1.01)^*^	0.60 (0.25–1.46)
**Condom use in past 6 months**				
Never	91 (41.5)	128 (58.5)	Ref	Ref
Sometimes	207 (38.4)	332 (61.6)	0.88 (0.64–1.21)	1.11 (0.71–1.75)
Always	332 (65.7)	173 (34.3)	2.70 (1.95–3.74)^*^	4.14 (2.34–7.33)^*^
**Used condoms in last sexual encounter**				
No	267 (44.0)	340 (56.0)	Ref	Ref
Yes	411 (57.0)	310 (43.0)	1.69 (1.36–2.10)^*^	0.91 (0.62–1.34)
**Carry condoms when needed**				
No	185 (32.6)	382 (67.4)	Ref	Ref
Yes	583 (67.4)	282 (32.6)	0.23 (0.19–0.29)^*^	0.17 (0.12–0.23)^*^
**Refuse sex no condom**				
Unlikely	208 (60.6)	135 (39.4)	Ref	Ref
Likely	577 (52.1)	530 (47.9)	0.71 (0.55–0.90)^*^	0.36 (0.25–0.53)^*^
**Suggest condom to partner**				
No	51 (47.2)	57 (52.9)	Ref	Ref
Yes	710 (54.0)	604 (46.0)	1.31 (0.89–1.95)	2.09 (1.08–4.04)^*^
**Discuss HIV testing**				
No	210 (66.2)	107 (33.8)	Ref	Ref
Yes	632 (54.9)	520 (45.1)	0.62 (0.48–0.80)^*^	0.86 (0.58–1.29)
**Worry about HIV**				
Not worried at all	258 (45.4)	310 (54.6)	Ref	Ref
Worried	175 (56.8)	133 (43.2)	1.58 (1.19–2.09)^*^	2.02 (1.33–3.05)^*^
Very worried	445 (67.1)	218 (32.9)	2.45 (1.95–3.01)^*^	3.84 (2.68–5.48)^*^

* *Significant at p < 0.05*.

^**^University students is the reference group (ref = 0).

Out of school youth had a 45% probability of having multiple sexual partners (AOR = 0.54, 95% CI: 0.38–0.80) compared to university students. With respect to condom use, the odds of always using a condom (AOR = 4.14, 95% CI: 2.34–7.33), and suggesting the use of a condom (AOR = 2.1, 95% CI: 1.08–4.0) were higher for university students than out of school youth. University students were also four times more likely to be very worried about HIV transmission than out of school youth (AOR = 3.83, 95% CI: 2.68–5.48).

### Factors Associated With Partner Notification

Bivariate and multivariable logistic regression analyses were done to examine the effect of the sociodemographic and behavioral characteristics on the decision to notify the partner when diagnosed with an STI. Age, sex, educational group, number of sexual partners, number of years in the relationship, transactional sex, chances of HIV testing with partner, and condom use in last sexual encounter were selected through an iterative process of variable selection. In the reduced model, sex, educational group, and chances of HIV testing with partner were found to have an association with partner notification (Table 5).

**Table 5:** Bivariate and multivariable logistic regression analysis of sociodemographic and behavioral characteristics associated with partner notification.

	**Notified partner**			
	**Yes *n* (%)**	**No *n* (%)**	**UOR (95% CI)**	**AOR (95% CI)**
**Age**				
18–24	79 (73.2)	29 (26.8)	Ref	
25–35	15 (71.4)	6 (28.6)	0.92 (0.32–2.59)	0.70 (0.14–3.55)
**Sex**				
Female	78 (77.2)	23 (22.8)	Ref	Ref
Male	16 (59.3)	11 (40.7)	0.43 (0.17–1.05)	0.17 (0.03–0.87)^*^
**Education group**				
Out of school	72 (76.6)	22 (23.4)	Ref	Ref
University student	22 (62.9)	13 (37.1)	0.52 (0.22–1.19)	0.18 (0.04–0.74)^*^
**Number of sexual partners in the last 12 months**				
One	55 (77.5)	16 (22.5)	Ref	Ref
Two or more partners	33 (68.8)	15 (31.2)	0.67 (0.28–1.46)	1.79 (0.38–8.33)
**Number of years in relationship**				
<1	9 (52.9)	8 (47.1)	Ref	Ref
1–4	51 (82.3)	11 (17.7)	4.12 (1.30–13.07)^*^	1.09 (0.22–5.45)
≥5	26 (81.2)	6 (18.8)	3.85 (1.05–14.16)^*^	1.13 (0.15–8.49)
**Had transactional sex in past 12 months**				
No	90 (75.6)	29 (24.4)	Ref	Ref
Yes	3 (37.5)	5 (62.5)	0.19 (0.43–0.86)^*^	0.60 (0,45–8.27)
**Used condoms in last sexual encounter**				
No	53 (70.7)	22 (29.3)	Ref	Ref
Yes	40 (78.4)	11 (21.6)	1.51 (0.66–3.47)	1.57 (0.48–5.13)
**HIV partner testing**				
Unlikely	17 (45.9)	20 (54.1)	Ref	Ref
Likely	75 (83.3)	15 (16.7)	5.88 (2.51–13.51)^*^	12.51 (3.04–51.45)^*^

* *Significant at p < 0.05*.

^**^Ref and Ref are reference groups for UOR and AOR.

The probability of notifying a partner about an STI infection was 82% among university students compared to their counterparts (AOR = 0.18, 95% CI: 0.04–0.74). Male participants had an 83% probability to notify their partners (AOR = 0.17, 95% CI: 0.03–0.87) compared to females. The odds of notifying a partner was 1.79 times more for those having multiple sexual partners than those who had only one partner (AOR = 1.79, 95% CI: 0.38–8.33). The probability of notifying a partner was at a low of 40% among those who had transactional sex (AOR = 0.60; 95% CI: 0. 45–8.27) than those who had not engaged in transactional sex. Those who were likely to test for HIV with a partner were 12 times more likely to notify their partners as compared to those who would not (AOR = 12.51, 95% CI: 3.04–51.45).

## Discussion

We report on the differences in self-reported risky sexual behaviors, STI health-seeking behaviors, and STI partner notification practices of university students and out of school youth. The study findings provide an insight into the differences of sexual health matters between the out-of-school youth who live in a peri-urban community and the university students who are mainly based in the university residence within the same community.

Being in casual sexual relationships and changing a sexual partner in the past 12 months was significantly prevalent among the university students. Studies conducted in Ethiopia and Nigeria on different youth groups such as those in higher learning institutions as well as those who are in the home environment being influenced by family and living with both parents described that casual sexual relationships for those among university students are influenced by the context of large numbers of young people living together, interacting and engaging in sexual experimentation and for those with adequate family support and living with both parents have been positively associated with protective sexual behaviors (23, 46). Other studies attributed casual sex among the population of university students to the general permissive attitude regarding premarital sex among young people, the liberal nature of campus life, students' independence from parental control, transition from adolescence into adulthood, and the tertiary institution restrictions that give the students the responsibility for their own behavior (24, 47–49). The findings of the current study showed that a significantly high proportion of out of school youth had steady partners and long-term relationships. However, on the other hand, high proportion of the out of school youth had more multiple sexual partners and had more frequent transactional sex in the past year. Similar findings were reported in other studies, where the behavior of multiple concurrent partners among the out-of-school youths was cited to stem from the context of being in an unprotected environment, not having specific goals such as educational attainment and having low health literacy compared to the university students (50). Other studies attributed the sexual behaviors of out of school youth to disappointments and financial strain, especially among the unemployed vulnerable young women (51, 52).

We found that a significantly large proportion of out of school youth did not use condoms during the last sexual intercourse compared to university students. They also reported low condom use in the past 6 months, and had low HIV transmission risk perception, while university students were four times more likely to always use condoms, twice more likely to suggest condom use to a sexual partner, and three times more likely to have the high HIV risk perceptions. The low level or inconsistent condom use and low HIV transmission risk perceptions are associated with being in a long-term sexual relationship and trusting the sexual partner, while being single, having casual sex, and not living with a sexual partner is associated with consistent condom use, and condom negotiation self-efficacy (53–57). The studies cited above confirm that self-reported inconsistent condom use and being in a steady sexual relationship were common among the out of school youth compared to the university students. Furthermore, the high proportion of casual sexual relationships among the university students explains the reluctance to discuss HIV testing and to consider STI PN with the casual partner.

Of the 124 participants who reported to have been diagnosed with STIs, a significantly high proportion were the out of school youth (85 vs. 15%). They also had low health seeking behaviors for STI diagnosis and treatment as only a few received treatment. With respect to PN, out of school youth had a low PN rate compared to the university students (69 vs. 89%). There is a dearth of literature comparing PN behaviors of out-of-school youth and university students, but literature among the adult population in general relates the low STI treatment seeking behavior and low PN to stigma. The fear of stigma and rejection have been cited as the barriers to notify sexual partners about an STI diagnosis, reporting STI symptoms, taking and completing treatment, and abstaining from sexual intercourse during the period of treatment. As it is prescribed in the syndromic management of STIS regime, the fear of stigma might explain the high proportion of both university and the out-of-school youth having sexual intercourse during STI treatment (33, 34). The likelihood of notifying a partner about an STI infection was higher among those who would take an HIV test together with a sexual partner, suggesting that discussing about an HIV test reduces the stigma surrounding HIV and STIs.

Regarding the preferred type of STI partner notification, both groups reported to have preferred face-to-face notification and the PN slips, followed by provider initiated notification by SMS from the clinic. These findings illustrate the low acceptability of PN using the slip which is the only modality that is offered in the syndromic management of the protocols and guidelines for STIs in South Africa. We further found that the PN slip was less preferred, while face-to-face followed by SMS PN notification modalities were more preferred by those who reported not to have been diagnosed with STIs. The preference for face-to-face PN poses a major challenge with the target group in this study since university students have casual partners while the out of school youth have steady partners with some of them having transactional sex. This suggests that both groups did not appreciate the feasibility of getting hold of all their partners. The appropriateness of the PN slip is questionable because of the low level of acceptability among other high risk groups, and the fact that both groups are involved in casual, multiple, and concurrent relationships (35). These behaviors pose a challenge to the participants in reporting and submitting the accurate number of PN slips to the sexual contacts and/or partners.

A high proportion of the university students reported that they would not find it easy to deliver a PN slip to their partners comparted to the out of school youth. This could be explained by the fact that university students are mainly in casual relationships, while the uneasiness of delivering the PN slip among the out of school youth who mostly reported to be in steady relationships could be out of fear of introducing doubt and conflict into their relationships. These possibilities have been cited in the literature (33, 35). Of interest is that the university students reported that even though they would not find it easy to deliver the slips to their sexual partner, they were more likely to submit the notification slip while their counterpart reported that they would prefer to receive a notification slip from a partner.

## Conclusion

This study presents a comparative analysis of sexual behaviors, self-reported STI diagnosis, health seeking behavior, and preferred PN methods of university students and out of school youth. There are some similarities and differences in the types of risky sexual behaviors practiced across the two groups. University students reported more casual relationships and sex however with higher condom use as compared to out of school youth. STI self-reported prevalence in this study cannot be generalized to the population however worth noting is that despite out of school youth being in more steady relationship with fewer reports of casual sex, they had higher proportions of being diagnosed with STI in the past 12 months than university students. This study indicates a greater need to dedicate research on impact of varying contextual factors that could be associated with the reported risky sexual behavior, STI diagnosis and preferred health interventions. STI partner notification was acceptable for both groups however different methods were preferred across both groups, however with the least preferred method being PN slips which is the current method used (58). Programme developers in the STI prevention programmes should consider adding SMS notification to the current PN slip. Furthermore, there is a need for developing health promotion scripts on disclosing STIs to sexual partners to empower the majority of the youth who prefer face-to-face PN over the other two methods.

### Study Limitations

This study was limited, first, by the fact that it was based on self-reports and, thus, subject to elements of response bias due to the sensitive nature of discussions around sex. Second, there were potentials for recall bias since respondents were expected to provide information on behaviors committed in the past. Different sampling methods were adopted for the two groups and this could have introduced selection bias, and because student from University were from Health science university their risk perception and behavior may be higher than those out of school. Random sampling among University students reduced the bias but the sensitivity of the questions on sexual behavior would elicit socially desirable responses for both the groups. Using anonymous, self-administered questionnaires provided confidentiality and hence could have reduce socially desirable responses. Convenient sampling of the out-of-school-youth introduced selection bias since only interested youth came forth.

Nevertheless, the findings have several implications and benefits for the design of STI control and prevention among the youth in communities and university campuses.

## Data Availability Statement

The original contributions presented in the study are included in the article/supplementary files, further inquiries can be directed to the corresponding author.

## Ethics Statement

The studies involving human participants were reviewed and approved by the Research Ethics Committee of Sefako Makgatho Health Sciences University (SMUREC/H/284/2015: IR). The patients/participants provided their written informed consent to participate in this study.

## Author Contributions

SM and MM: conceptualization, methodology, investigation, data curation, project administration, and funding acquisition. SM: validation. MM and NH: formal analysis and writing—original draft preparation. MM: resources and writing review and editing. NH: visualization. All authors have read and agreed to the published version of the manuscript.

## Funding

This research was funded by VLIR/UOS, grant number ZIUS2015AP021 and NRF CSUR150728131833. The APC was funded by School of Transdisciplinary Research and Graduate Studies; College of Graduate Studies, UNISA, Muckleneuk, Pretoria, 0001.

## Conflict of Interest

The authors declare that the research was conducted in the absence of any commercial or financial relationships that could be construed as a potential conflict of interest.

## Publisher's Note

All claims expressed in this article are solely those of the authors and do not necessarily represent those of their affiliated organizations, or those of the publisher, the editors and the reviewers. Any product that may be evaluated in this article, or claim that may be made by its manufacturer, is not guaranteed or endorsed by the publisher.
